# Synthesis and Application of Amine Functionalized Iron Oxide Nanoparticles on Menaquinone-7 Fermentation: A Step towards Process Intensification

**DOI:** 10.3390/nano6010001

**Published:** 2015-12-25

**Authors:** Alireza Ebrahiminezhad, Vikas Varma, Shuyi Yang, Younes Ghasemi, Aydin Berenjian

**Affiliations:** 1 Noncommunicable Diseases Research Center, Fasa University of Medical Sciences, Fasa 74615, Iran; a_ebrahimi@sums.ac.ir; 2 Department of Pharmaceutical Biotechnology, School of Pharmacy and Pharmaceutical Sciences Research Center, Shiraz University of Medical Sciences, Shiraz 71348, Iran; ghasemiy@sums.ac.ir; 3 School of Engineering, Faculty of Science and Engineering, The University of Waikato, Hamilton 3240, New Zealand; v_p@hotmail.co.nz (V.V.); missshuyi@hotmail.com (S.Y.)

**Keywords:** immobilization, downstream processing, magnetic nanoparticles, MK-7, bioseparations, fermentation

## Abstract

Industrial production of menaquione-7 by *Bacillus subtilis natto* is associated with major drawbacks. To address the current challenges in menaquione-7 fermentation, studying the effect of magnetic nanoparticles on the bacterial cells can open up a new domain for intensified menqainone-7 process. This article introduces the new concept of production and application of l-lysine coated iron oxide nanoparticles (l-Lys@IONs) as a novel tool for menaquinone-7 biosynthesis. l-Lys@IONs with the average size of 7 nm were successfully fabricated and were examined in a fermentation process of l-Lys@IONs decorated *Bacillus subtilis natto*. Based on the results, higher menaquinone-7 specific yield was observed for l-Lys@IONs decorated bacterial cells as compared to untreated bacteria. In addition, more than 92% removal efficacy was achieved by using integrated magnetic separation process. The present study demonstrates that l-Lys@IONs can be successfully applied during a fermentation of menaquinone-7 without any negative consequences on the culture conditions. This study provides a novel biotechnological application for IONs and their future role in bioprocess intensification.

## 1. Introduction

Menaquinone-7 (MK-7) plays a key role in reducing health disorders such as cardiovascular disease, osteoporosis, diabetes, Alzheimer’s disease, and liver, blood and prostate cancers [1]. MK-7 can only be produced thorough a fermentation process, mainly through a metabolic pathway of *Bacillus subtilis* species. However, this valuable extracellular compound is not readily accessible due to the significant barriers in the production process such as low vitamin yield through the bacterial metabolic pathway, long fermentation period, and several tedious and inefficient operation units (more than 20 different steps) [2]. Therefore, to address these challenges there is a need for sustainable production methods and technologies [3].

Process intensification as a method for decreasing the process steps can be a promising approach. These reductions can come from decreasing the size of individual equipment or from removing the number of involved unit operations [4]. Process integration, such as *in-situ* cell recovery, has introduced as a valuable tool to reduce the operation units and increase the yield of process. Continuous separation of product and microorganisms from the bioreactor by adsorption of the target using functionalized surfaces significantly reduces the production limitations. These techniques can bypass the need for several purification steps such as filtration, centrifugation or extraction before final purification is performed [5]. Bioprocess intensification often has been focused on decreasing the number of bioseparation steps. Much work has been done on the use of direct capture methods such as expanded bed adsorption and high gradient fishing to recover the product directly from a crude fermentation broth; however, these approaches encounter significant drawbacks and are challenged.

Nanoparticles due to their unique physicochemical properties can play various applications at process modification and intensification. Therefore, the association of nanotechnology and biotechnology is expected to solve several biological problems. Among the nanoparticles, Iron Oxide Nanoparticles (IONs) have been extensively used in the biological sciences for cell labeling, RNA and DNA purification and enzyme and protein immobilization [6]. Recently, IONs have been used for bacterial cells immobilization and separation [7]. Surface of bacterial cells can be simply decorated with IONs by electrostatic and hydrophobic interactions. Decorated bacteria show a significant response to magnetic field and easily can be separated by applying a magnetic field [8]. As compared to centrifugation, this approach has significant advantages while allowing for the reusability of bacteria. The common immobilization techniques are based on imbedding the cells in a polymeric matrix like calcium alginate. This matrix acts like a barrier for mass transfer and put the cells in a microenvironment, which is different from fermentation media. Immobilization with magnetic nanoparticles would not make such a barrier around microorganisms and combines the advantages of cell immobilization with those of free cell fermentation [8]. This novel technique allows for high product purity in only one step and minimizing the overall process costs [5]. Magnetic immobilization can bypass the need for several purification steps before final purification and packaging.

However, naked IONs do not have a sufficient physicochemical stability and are toxic to microorganism [9,10,11,12,13]. These detrimental properties could be significantly eliminated by the use of biocompatible coatings [14]. Amino acids due to their chemical simplicity, surface activity, and biocompatibility can be an appropriate coating for designing a next generation of intensified bioprocesses. l-lysine (l-Lys) coating has no undesirable effect on the main characteristics of IONs and also introduces amine functional groups to the nanoparticles [14]. Amine functionalization would improve particles interaction with large negatively charged cell membrane domains and hence increase the chance of surface interactions. On the other hand, synthesis of l-Lys coated IONs could be done in a one pot aqueous reaction [9,10,14,15,16,17]. This simple synthesis pathway and lack of organic solvents are among the main advantages of l-lysine coatings.

The aim of the present study is, therefore, to address the current issues in the production and recovery of MK-7. The hypotheses were to synthesis l-Lys@IONs and investigate their effect on *Bacillus subtilis natto* growth, MK-7 production, and the possibility of designing a fermentation process with magnetically immobilized cells for *in-situ* product and cell recovery.

## 2. Experimental Section

### 2.1. Materials

FeCl_3_·6H_2_O, FeSO_4_·4H_2_O, l-Lys, methanol, 2-propanol and *n*-hexane were obtained from Sigma–Aldrich (St. Louis, MO, USA). Pure MK-7 (99.3%) was purchased from ChromaDex (Boulder, CO, USA) for calibration and HPLC analysis. Soy peptone, glycerol and yeast extract were obtained from BD (Becton-Dickinson Co., Franklin Lakes, NJ, USA).

### 2.2. Synthesis and Characterization of l-Lys@IONs

Briefly, FeSO_4_·4H_2_O (0.6 g), FeCl_3_·6H_2_O (1.17 g) and l-Lys (1.6 g) with the molar ratio of 1:1.75:4 were dissolved in 50 mL distilled water. Under N_2_ atmosphere at 70 °C and continuous stirring, ammonium hydroxide (5 mL, 32%) was added to the mixture. After 1.5 h, the black precipitate was harvested using a magnetic field, washed three times with boiling water and dried in an oven (50 °C) overnight. The prepared particles were characterized using Transmission Electron Microscopy (TEM, Philips, CM 10; HT 100 kV, Philips Electron Optics, Eindhoven, The Netherlands), Fourier Transformed Infrared spectroscopy (FTIR, Bruker, Vertex 70, FT-IR Spectrometer, Bruker, Kassel, Germany), Differential Scanning Calorimetry (DSC, Thermoanalyser DSC 302, TA Instruments, New York, NY, USA), Vibrating Sample Magnetometer and X-Ray powder diffraction spectroscopy (Siemens AG, Munich, Germany).

### 2.3. Immobilization of Bacterial Cells with l-Lys@IONs and Fermentation

*Bacillus subtilis natto* cells [18] were cultured in tryptic soy broth and cells were harvested and washed with normal saline. The cells were suspended in normal saline and mixed with various concentrations of l-Lys@IONs. The mixtures were incubated in a shaker incubator (150 rpm, 37 °C) for attachment of nanoparticles to the cells surface. After incubation for 15 min, immobilized cells were transferred to fermentation media consisting of 1% (*w*/*v*) yeast extract, 5% (*w*/*v*) glycerol, 1.5% (*w*/*v*) soy peptone. All the fermentation experiments were conducted at 40 °C for a period of five days. A magnetic field (Neodymium magnet: 800 gauss) was used for cell separation process studies. Statistical significant was determined by analysis of variance (ANOVA) and was accepted at *p* < 0.05.

### 2.4. MK-7 Extraction and Measurement Procedure

MK-7 was extracted from the fermentation media using 2-propanol and *n*-hexane with the ratios of (1/2, *v*/*v*) and 1/4 (liquid/organic, *v*/*v*) [19]. In each experiment, after the addition of organic solution, sample was vigorously shaken with a vortex, followed by centrifugation at 3000 rpm for 10 min afterwards. The organic layer was then collected from the aqueous layer to recover the extracted MK-7. High Performance Liquid Chromatography (HPLC, Waters Co., Bedford, MA, USA) with a photon diode array UV detector was used for the analysis of MK-7 concentration. Samples were separated by C18 Gemini column (5 μm, 250 × 4.6 mm, Phenomenex Co., Torrance, CA, USA) at 40 °C. The mobile phase consisted of methanol that was used at a flow rate of 1 mL/min.

## 3. Results and Discussion

**Figure 1:**
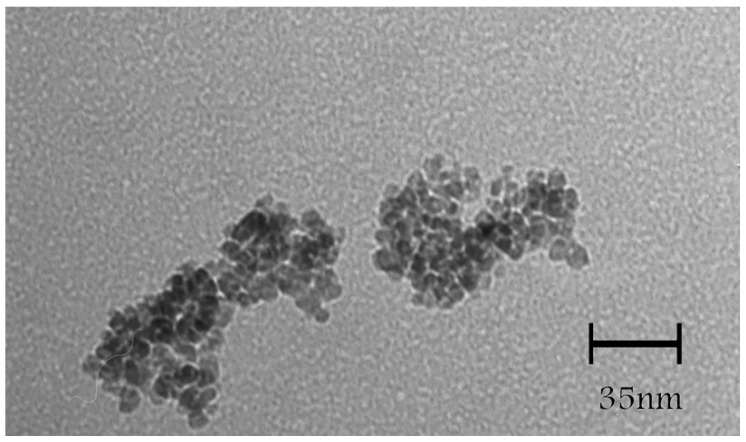
Transmission electron micrographs of l-lysine coated magnetite nanoparticles.

**Figure 2:**
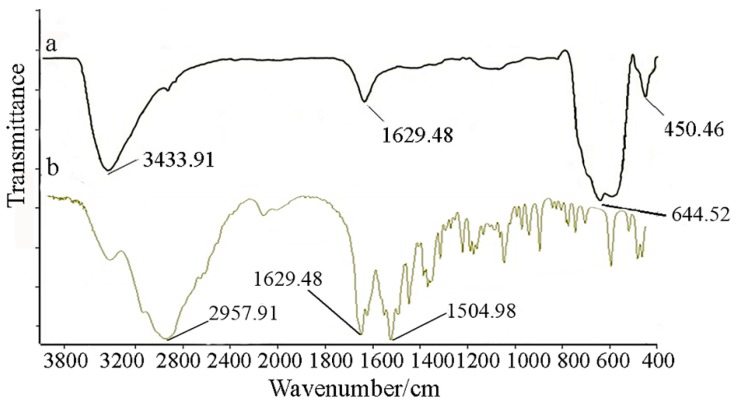
Fourier transform infrared spectroscopy (FTIR) spectra of (**a**) l-lysine coated magnetite nanoparticles and (**b**) pure l-lysine.

**Figure 3:**
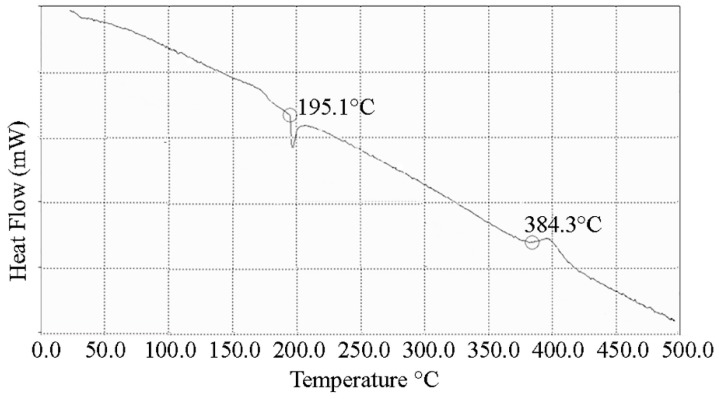
Differential scanning calorimetry (DSC) curves of l-lysine coated magnetite nanoparticles.

**Figure 4:**
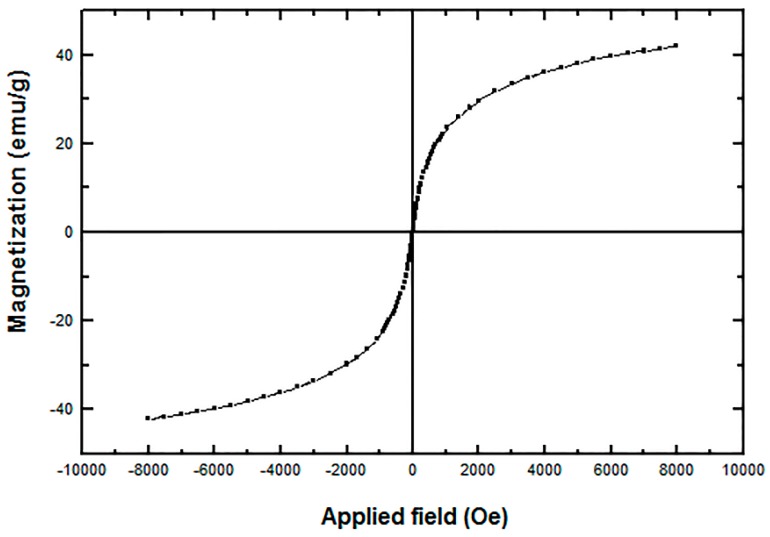
Vibrating sample magnetometer (VSM) diagrams of l-lysine coated magnetite nanoparticles.

**Figure 5:**
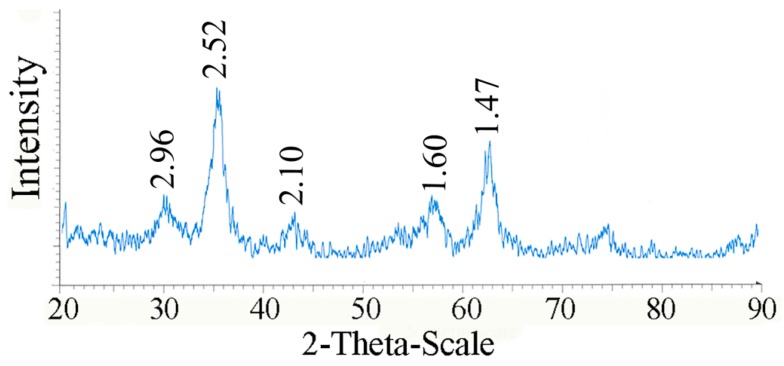
X-ray power diffraction patterns of l-lysine coated magnetite nanoparticles.

TEM micrographs of the l-Lys@IONs showed that the prepared nanoparticles are fairly uniform having a narrow particles size distribution ranging from 4 to 10 nm with the average size of 7 nm (Figure 1). The FTIR spectrum of l-Lys@IONs is presented in Figure 2a. The Fe–O characteristic peaks of magnetite nanoparticles were appeared at about 637 cm^−1^ and 450 cm^−1^, respectively. In the aqueous medium, the surface of magnetite nanoparticles was modified by OH groups, due to coordination of unsaturated Fe atoms with hydroxyl ions or water molecules. These OH groups absorb IR waves at about 3400 cm^−1^ (stretching) and 1630 cm^−1^ (deforming) [14]. In addition, C–O and C=O stretching vibrations are apparent at ~1439 cm^−1^ and ~1630 cm^−1^, respectively. The peak at 2921 cm^−1^ is due to CH stretching vibration and N–H stretching vibration overlays with OH stretching at 3419 cm^−1^. Compared to pure l-Lys spectrum (Figure 2b), shortening of the carboxyl group’s peak in l-Lys@IONs is due to interaction with OH groups at the surface of the nanoparticles [14]. DSC curves of l-Lys@IONs are presented in Figure 3. An endothermic peak, due to oxidation and change in crystallinity of Fe_3_O_4_ crystals, can be seen at 195.1 °C. Decomposition of l-lysine coating occurred at about 384.3 °C and produced an exothermic peak [10,15,16]. Saturation magnetization analysis results are depicted in Figure 4. No hysteresis was seen and magnetization curves were completely reversible exhibiting the super paramagnetic behavior of the produced particles. X-ray power diffraction patterns of the nanoparticles are validated by the characteristic features of magnetite nanoparticles having intensity peaks at 2θ degrees of 30°, 35.5°, 43°, 57°, and 63° (Figure 5).

**Figure 6:**
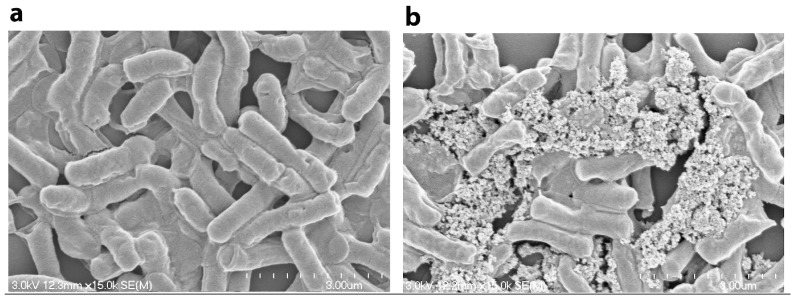
SEM image of the produced *Bacillus subtilis natto* cells (**a**) untreated and (**b**) decorated with l-lysine-IONs.

IONs with amino acid coating have significantly smaller size than bacterial cells, and the high surface/volume ratio of these nanoparticles would offer great surface area for attachment onto cell surfaces. Once these functionalized particles mixed with bacterial cells, the nanoparticles could be attached and deposited onto the surfaces of cells by hydrogenic bonds and electrostatic or hydrophobic interactions [8,10]. The clusters of bacterial cells and IONs then could be formed rapidly [20]. Figure 6 illustrates the successful entrapment and immobilization of *Bacillus subtilis natto* cells in nanoparticle clusters as compared to untreated cells.

**Figure 7:**
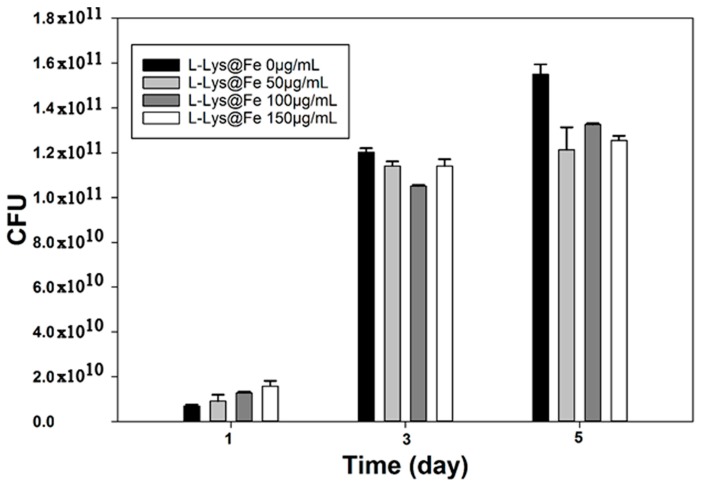
*Bacillus subtilis natto* cells growth at different at l-lysine-IONs concentrations.

The effects of various concentrations of l-Lys@IONs on the growth of *Bacillus subtilis natto* cells are presented in Figure 7. As compared to free-floating bacteria, attachment of the fabricated nanoparticles on bacterial cells resulted in approximately 16% growth inhibition (*p <* 0.05). At the end of fermentation (day five), the control sample reached 1.54 × 10^11^ CFU/mL, whereas cell density for bacterial cells exposed to 50, 100 and 150 μg/mL l-Lys@IONs were 1.21 × 10^11^, 1.33 × 10^11^ and 1.25 × 10^11^ CFU/mL, respectively. However, there were no significant cell growth differences among the treated samples with different l-lysine concentrations (*p* > 0.05). Different bacterial species exhibited different susceptibilities to nanoparticles. Similar to our results, some investigations have reported a growth inhibitory effect of IONs on strains including *Staphylococcus aureus*, *Pseudomonas aeruginosa*, *Escherichia coli* and *Listeria monocytogenes* [9,11,12,13]. On the other hand, there is evidence that IONs exhibited a dose dependent stimulatory effect on the microbial growth in case of *Klebsiella pneumoniae, Pseudomonas aeruginosa, Enterococcus faecalis* and *Candida albicans* strains [12]. Release of free iron from the IONs could catalyze production of reactive oxygen species (ROS) in the Fenton’s reaction and ROS can damage the cells [9]. There is a nanoparticle specific mechanism that is due to stress or stimuli from physical properties of IONs such as surface, size and shape [21]. Nonspecific interactions with membrane compounds have been reported to result in disorganization of lipid packing in the microorganism membrane. Such an effect may cause loss of membrane transport selectivity [8,22].

In some cases, free iron or iron ions that are released from the IONs can be used as a source of iron and enhance the cells growth rate [16]. It might be suggested that the differences, which are observed in the antimicrobial activity of these magnetic particles, reflect differences between microbial cell walls. Moreover, different factors such as synthesis procedure, shape, size and composition of the particles can lead to different conclusions even for very closely related nanostructures [23].

**Figure 8:**
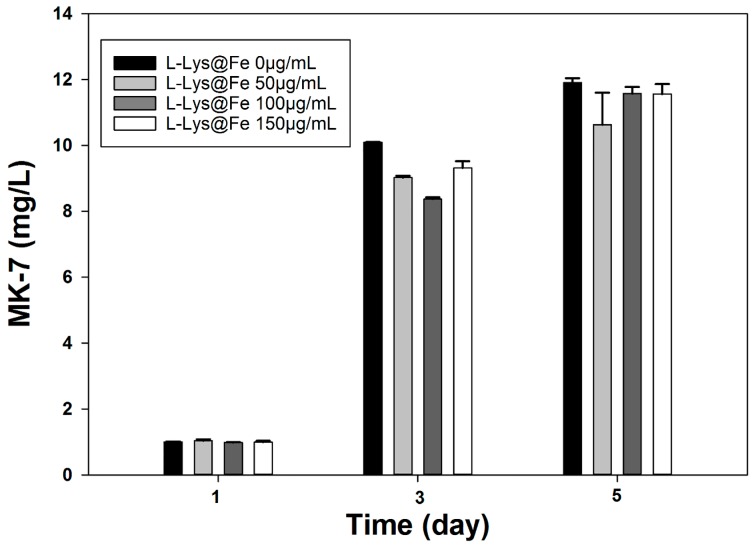
MK-7 production at different at l-lysine-IONs concentrations.

As can be seen in Figure 8, presence of IONs showed no negative effect on *Bacillus subtilis natto* metabolic activity and consequently MK-7 production. MK-7 concentration was enhanced in a time dependent manner during the fermentation period. MK-7 concentration reached the highest level of 11.8 ± 0.14 mg/L while using the free-floating *Bacillus* cells. Additionally, MK-7 production were 10.8 ± 0.91, 11.57 ± 0.12 and 11.56 ± 0.31 mg/L for the cells decorated with 50, 100 and 150 μg/mL l-Lys@IONs, respectively. There were no statistically significant differences between the MK-7 production among the investigated samples (*p* > 0.05). The majority of MK-7 is produced during the bacterial growth phase and only 20% of total MK-7 is generated during the stationary phase [24].

To further investigate the impact of immobilization on the MK-7 production, specific yield (SY) was calculated based on Equation (1).

SY = MK-7 Concentration/CFU
(1)

**Figure 9:**
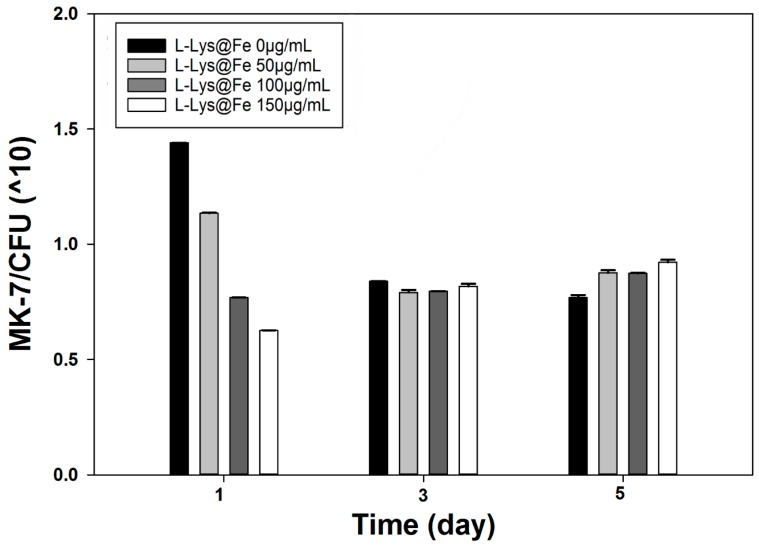
MK-7 specific yield at different at l-lysine-IONs concentrations.

As shown in Figure 9, presence of l-Lys@IONs resulted in significantly higher SY as compared to untreated and free-floating samples (*p* < 0.05). It has been reported that decoration of bacterial cells surface with IONs makes the cells more metabolically efficient [8]. A possible mechanism for this enhancement is that the bounded nanoparticles to bacterial surface make the cell membranes more permeable and facilitate mass transfer via cell barriers. The added IONs diffuse to the surface of the membrane and are presumably adsorbed and diffuse within the membrane; step by step the membrane permeability is increased [8].

All l-Lys@IONs treated samples were further investigated for the recovery and reusability. A Neodymium magnet (800 gauss) was used for precipitation and separation studies. The separation studies on *Bacillus subtilis natto* cells showed a dose-dependent increase in the number of captured microorganisms, namely 77% (50 µg/mL l-Lys@IONs), 88% (100 µg/mL l-Lys@IONs), and 92% (150 µg/mL l-Lys@IONs), with the possibility of running five successful recycle batches. This behavior could be ascribed to the stronger entrapment of bacterial cells in magnetic clusters of nanoparticles by increase l-Lys@IONs concentration. *In-situ* cell recovery has emerged as a valuable tool to increase the overall process efficacy and minimize the costs. Continuous separation of MK-7 (product) and microorganisms from the bioreactor by adsorption of the target using functionalized surfaces significantly reduces the production limitations. These can be proteolytic degradation, inhibition of target functionality and target production. Magnetic separation technology is scalable and can easily be integrated in a recycle loop in a bioreactor to achieve a rapid recovery of bacterial clusters. Intensified bioprocess by integrating MK-7 formation and *Bacillus subtilis natto* recovery can be achieved by the use of magnetized l-Lys@IONs. In addition, reduction in the number of process steps is also another advantage to reduce the interaction of the accumulated product with the system (e.g., product inhibition). By applying this technology, losses due to uncontrolled product damage, which can arise from reactions with substances present in the broth, will be diminished.

## 4. Conclusions

The results of this study demonstrate that l-lysine coated IONs can be used as a promising method for immobilization, MK-7 production and cell recovery during the fermentation. The *Bacillus subtilis natto* cells were effectively recovered from the culture media by using this novel method. The presence of l-Lys@IONs clusters were found to be beneficial to the overall MK-7 yield without showing any toxicity effects on *Bacillus subtilis natto* cells. This study shows great promise for fabrication and application of l-Lys@IONs in cell immobilization when the extracellular product is produced. It is, therefore, critical to consider the results of the present study for further development of an industrial level production of MK-7.

## References

[1] Berenjian A., Mahanama R., Kavanagh J., Dehghani F. (2015). Vitamin K series: Current status and future prospects. Crit. Rev. Biotechnol..

[2] Berenjian A., Mahanama R., Talbot A., Regtop H., Kavanagh J., Dehghani F. (2014). Designing of an intensification process for biosynthesis and recovery of menaquinone-7. Appl. Biochem. Biotechnol..

[3] Wohlgemuth R. (2009). The locks and keys to industrial biotechnology. New Biotechnol..

[4] Vaghari H., Eskandari M., Sobhani V., Berenjian A., Song Y., Malmiri H.J. (2015). Process intensification for production and recovery of biological products. Am. J. Biochem. Biotechnol..

[5] Marques M.P.C., Fernandes P. (2011). Microfluidic devices: Useful tools for bioprocess intensification. Molecules.

[6] Can K., Ozmen M., Ersoz M. (2009). Immobilization of albumin on aminosilane modified superparamagnetic magnetite nanoparticles and its characterization. Colloid Surf. B.

[7] Liu X., Guan Y., Yang Y., Ma Z., Wu X., Liu H. (2004). Preparation of superparamagnetic immunomicrospheres and application for antibody purification. J. Appl. Polym. Sci..

[8] Ansari F., Grigoriev P., Libor S., Tothill I.E., Ramsden J.J. (2009). DBT degradation enhancement by decorating Rhodococcus erythropolis IGST8 with magnetic Fe_3_O_4_ nanoparticles. Biotechnol. Bioeng..

[9] Ebrahiminezhad A., Davaran S., Amini S.R., Barar J., Moghadam M., Ghasemi Y. (2012). Synthesis, characterization and anti-Listeria monocytogenes effect of amino acid coated magnetite nanoparticles. Curr. Nanosci..

[10] Ebrahiminezhad A., Amini S.R., Davaran S., Barar J., Moghadam M., Ghasemi Y. (2014). Impacts of iron oxide nanoparticles on the invasion power of Listeria monocytogenes. Curr. Nanosci..

[11] Chatterjee S., Bandyopadhyay A., Sarkar K. (2011). Effect of iron oxide and gold nanoparticles on bacterial growth leading towards biological application. J. Nanobiotechnol..

[12] Grumezescu A.M., Mihaiescu D.E., Mogoşanu D.E., Chifiriuc M.C., Lažr V., Čluǧrescu I., Třistaru V. (2010). *In vitro* assay of the antimicrobial activity of Fe_3_O_4_ and CoFe_2_O_4_/oleic acid–core/shell on clinical isolates of bacterial and fungal strains. Optoelectron. Adv. Mat..

[13] Ramteke C., Sarangi B.K., Chakrabarti T., Mudliar S., Dewanand S., Pandey R.A. (2010). Synthesis and broad spectrum antibacterial activity of magnetite ferrofluid. Curr. Nanosci..

[14] Ebrahiminezhad A., Amini S.R., Davaran S., Barar J., Ghasemi Y. (2012). Impact of amino-acid coating on the synthesis and characteristics of iron-oxide nanoparticles (IONs). Bull. Kor. Chem. Soc..

[15] Ebrahiminezhad A., Ghasemi Y., Rasoul-Amini S., Barar J., Davaran S. (2013). Preparation of novel magnetic fluorescent nanoparticles using amino acids. Colloids Surf. B Biointerfaces.

[16] Ebrahiminezhad A., Amini S.R., Kouhpayeh A., Davaran S., Barar J., Ghasemi Y. (2015). Impacts of amine functionalized iron oxide nanoparticles on HepG2 cell line. Curr. Nanosci..

[17] Gholami A., Rasoul-amini S., Ebrahiminezhad A., Seradj S.H., Ghasemi Y. (2015). Lipoamino acid coated superparamagnetic iron oxide nanoparticles concentration and time dependently enhanced growth of human hepatocarcinoma cell line (Hep-G2). J. Nanomater..

[18] Mahanama R., Berenjian A., Valtchev P., Talbot A., Biffin R., Regtop H., Dehghani F., Kavanagh J.M. (2011). Enhanced production of menaquinone 7 via solid substrate fermentation from Bacillus subtilis. Int. J. Food Eng..

[19] Mahanama R., Berenjian A., Dehghani F., Kavanagh J. (2012). Modeling Menaquinone 7 production in tray type solid state fermenter. ANZIAM J..

[20] Larsen M.U., Seward M., Tripathi A., Shapley N.C. (2009). Biocompatible nanoparticles trigger rapid bacteria clustering. Biotechnol. Prog..

[21] Brunner T.J., Wick P., Manser P., Spohn P., Grass R.N., Limbach L.K., Bruinink A., Stark W.J. (2006). *In vitro* cytotoxicity of oxide nanoparticles: Comparison to asbestos, silica, and the effect of particle solubility. Environ. Sci. Technol..

[22] Epand R.M., Epand R.F., Savage P.B. (2008). Ceragenins (cationic steroid compounds), a novel class of antimicrobial agents. Drug News Perspect..

[23] Martin S.T., Morrison C.L., Hoffmann M.R. (1994). Photochemical mechanism of size-quantized vanadium-doped TiO_2_ particles. J. Phys. Chem..

[24] Berenjian A., Mahanama R., Talbot A., Biffin R., Regtop H., Valtchev P., Kavanagh J., Dehghani F. (2011). Efficient media for high menaquinone-7 production: Response surface methodology approach. New Biotechnol..

